# Identification and classification of reverse transcriptases in bacterial genomes and metagenomes

**DOI:** 10.1093/nar/gkab1207

**Published:** 2021-12-14

**Authors:** Fatemeh Sharifi, Yuzhen Ye

**Affiliations:** Luddy School of Informatics, Computing, and Engineering, Indiana University, Bloomington, IN 47408, USA; Luddy School of Informatics, Computing, and Engineering, Indiana University, Bloomington, IN 47408, USA

## Abstract

Reverse transcriptases (RTs) are found in different systems including group II introns, Diversity Generating Retroelements (DGRs), retrons, CRISPR-Cas systems, and Abortive Infection (Abi) systems in prokaryotes. Different classes of RTs can play different roles, such as template switching and mobility in group II introns, spacer acquisition in CRISPR-Cas systems, mutagenic retrohoming in DGRs, programmed cell suicide in Abi systems, and recently discovered phage defense in retrons. While some classes of RTs have been studied extensively, others remain to be characterized. There is a lack of computational tools for identifying and characterizing various classes of RTs. In this study, we built a tool (called myRT) for identification and classification of prokaryotic RTs. In addition, our tool provides information about the genomic neighborhood of each RT, providing potential functional clues. We applied our tool to predict RTs in all complete and draft bacterial genomes, and created a collection that can be used for exploration of putative RTs and their associated protein domains. Application of myRT to metagenomes showed that gut metagenomes encode proportionally more RTs related to DGRs, outnumbering retron-related RTs, as compared to the collection of reference genomes. MyRT is both available as a standalone software (https://github.com/mgtools/myRT) and also through a website (https://omics.informatics.indiana.edu/myRT/).

## INTRODUCTION

A reverse transcriptase (RT) is an enzyme that converts RNA into double stranded cDNA, and was discovered in 1970 in retroviruses (1). A well-known RT in retroviruses is HIV-1 RT, with DNA polymerase and RNase H enzymatic activities (2). All the retrotransposons (LTR and non-LTR) have RT genes (3). Bacterial RTs were first found in retrons retroelements. Bacterial RTs can also be found in bacterial defense systems against phages, e.g. in CRISPR-Cas systems and abortive infection systems (AbiA, AbiK and AbiP2) (4). RTs are also found in Diversity Generating Retroelements (DGRs) that facilitate tropism switching in phages, and accelerate the evolution of bacteria and archaea (5). Another important class of RTs can be found in mobile retroelements such as group II introns (GII/G2I) (6).

RTs involved in group II introns are the most abundant class of RTs in bacteria, and encode 57–75% of the bacterial RTs (4,6). Bacterial group II introns are self-splicing mobile elements, each consisting of a catalytic RNA and an intron-encoded protein (IEP) within the RNA. The IEP contains a RT domain and an X/thumb domain with maturase activity, along with DNA binding (D) and endonuclease (En) domains (4,7). Group II intron RTs and their template switching mechanisms are used in different gene and genome editing techniques including targetron and thermotargetron (8–11).

Retrons encode 12–14% of the bacterial RTs and are the second most frequent class of RTs (4,6). Although retrons were discovered three decades ago, their function remained unknown until recently that they were found to function as phage defense mechanisms (12,13). Bacterial retrons are non-LTR-retroelements that produce multicopy single-stranded DNAs (msDNAs). Most retrons consist of msr-msd sequence and a RT gene. Retrons can also encode toxin/antitoxin systems, which can be triggered or blocked by phage proteins (14,15). Retron RTs have been suggested as a tool for precise genome editing techniques (e.g. CRISPEY, SCRIBE and HiSCRIBE) as retrons can produce msDNA, and edit the target sequences (6,11,16,17).

RT genes are an essential component of Diversity Generating Retroelements (DGRs) (18). DGRs are found in bacteria, archaea and phages (19). DGRs are beneficial to the evolution and survival of their host; for instances, they can mediate tropism switching in Bordetella phage (20), mediate bacterial surface display (21), have a role in regulatory pathway tuning (22), and impact the underlying temperate phage-bacteria interactions in human gut microbiome (23,24). RTs in DGR systems are special, in the sense that they are error-prone. The RTs generate hyper-variable regions in specific target genes (e.g., genes encoding for tail fibre proteins and receptors), through a process called mutagenic retrohoming in which a template region (TR) is reversed transcribed into mutagenized cDNA (A-to-N mutations), and the mutagenized cDNA replaces a region (called variable region, VR) in the target gene which is similar to the TR region (5). Analyses of the target genes of DGR systems have shown that some pfam domains commonly encoded by target genes include, but are not limited to DUF1566, FGE-sulfatase, and Fib_succ_major (25).

RTs are also found in three types of abortive bacteriophage infection (Abi) systems including AbiA, AbiK and AbiP2 (4,26,27). Abi systems are a type of bacterial defense mechanisms that can lead to programmed death of a phage-infected cell, in order to protect the surrounding cell(s), and are often encoded by phages (e.g. P2 prophage of *E. coli*), and plasmids of bacterial genomes such as *Lactococcus lactis* (28). *L. lactis* has more than 22 different abortive infection systems (AbiA to AbiV) (29), among which, only two of them (AbiA and Abik) have a RT domain. Although AbiA and AbiK only share 23% identity, they both can stop phage P335 maturation by means of un-templated synthesis of a DNA covalently bonded to the reverse transcriptase domain in order to target the Rad52-like phage recombinases (30). C-terminal HEPN domain of AbiA (HEPN_AbiA_CTD), which may promote cell suicide through RNase activity, is fused to RT encoded by a gene found in an operon containing other genes including restriction modification system (RM system) (29).

RT genes are found in some classes of CRISPR-Cas (the bacterial adaptive immune) systems including several subsets of type III (III-A, III-B , III-C, and III-D) and type VI-A CRISPR-Cas systems that can acquire spacers directly from both DNA and RNA (31). RNA spacer acquisition has been used in methods such as Record-seq for transcriptional recording (11,32,33). CRISPR-Cas RTs are believed to have been emerged from multiple occasions: CRISPR-Cas RTs in archaea (Methanomicrobia) branch from class F of group II introns, CRISPR-Cas RT (ABX04564.1) in *Herpetosiphon aurantiacus* falls into the group II intron clade, CRISPR-Cas RT in *Haliscomenobacter hydrossis* is related to retron RTs (34). CRISPR-Cas RT in *Haemophilus haemolyticus* is related to AbiP2 RTs, and is associated with type I-C CRISPR-Cas systems (35). These are the examples showing association of Retron and AbiP2 RTs with CRISPR-Cas systems. *Streptomyces* spp. has several CRISPR-Cas RTs associated with type I-E CRISPR systems. As RNA activity is not common in type I CRISPR systems, experimental study of these CRISPR-Cas RTs may result in interesting findings (36).

Uncharacterized RTs are encoded by conserved ORFs in bacterial genomes, but their exact function and classification are unknown. Nevertheless, a few studies have suggested groupings of these RTs based on different criteria such as previously published data, sequence conservation of the RT motifs, and similarity of their fused protein motifs (4,6,37). The genomic neighborhood of these RTs can also provide us with information about the functions of these RTs: for instance, RTs of unknown classes 1 and 5 are fused with nitrilase motif in the C-terminal, RTs of unknown class 3 and class 8 tend to co-occur, unknown class 4 RTs have a fimbrial domain, and unknown class 10 of RTs have fused primase domain, suggesting a concerted priming and reverse by the protein that harbors these two domains (6). Despite the grouping, a few RTs remain unclassified as they don’t seem to have any close relationship with the other RTs in the collected dataset of RTs (6). A recent study, discovered that six classes of unknown RTs, including unknown class 3 and unknown class 8 are part of the defense systems against dsDNA phages (38)

Due to the importance and applicability of bacterial reverse transcriptases, there are tools and databases that have been developed for individual classes of RTs, or genetic elements that contain the RTs. There is a database of group II introns (http://webapps2.ucalgary.ca/groupii/) (39,40). MyDGR is a tool that we developed for identification of DGR systems and their associated RTs (5). However, a tool for characterization and classification of RTs remains lacking. We provide here the first pipeline for prediction of bacterial RTs and their classes, accompanied by genomic neighborhood information and visualizations. Furthermore, our pre-computed collection of putative RTs in all complete and bacterial genomes is easily accessible through myRT web-server at https://omics.informatics.indiana.edu/myRT/.

## MATERIALS AND METHODS

### Collection of the RT dataset

We curated a collection of 1,988 non redundant RTs based on different sources: CRISPR-Cas associated RTs were collected from (35), group II intron RTs were extracted from groupii (39), DGR RTs were previously collected as part of our research on DGRs systems (5) (which integrated DGR RTs from multiple sources (25,41–44)), nine AbiA (abortive_AbiA) representatives were downloaded from CDD (45), and RT sequences of the remaining classes were extracted from dataset of RTs collected by Toro *et al.* (4,35). As some of these datasets overlap, redundant RTs were removed (using cd-hit (46) cutoff value of 1). This integrated dataset contains RTs from group II introns (GII), CRISPR-Cas, DGRs, retrons, AbiA, AbiK, AbiP2, G2L, etc. This classification is mostly based on the grouping of RTs in (35), and was verified by us by analyzing the phylogenetic tree of RVT_1 motif sequences (see Results).

### Construction of class-specific HMMs of RVT_1 domain for RT prediction and classification

Since all RT sequences contain the RVT_1 domain (Pfam ID: PF00078), we used the RVT_1 motif sequences to build class specific Hidden Markov Models (HMMs) for RTs of different classes, which can then be used for identification and classification of RTs in genomes and metagenomes. For identification of RVT_1 domain in RT sequences, we used hmmscan (hmmer-3.2) (47) search against the Pfam-A model (PF00078) (48), and further validated the prediction using CDD-search (45) and manual check. Hits of low significance or with a short length were manually checked. We noticed that the predicted RT domains in UG2 RTs and 15 out of UG28 RTs were split into two fragments in these proteins, which resulted in poor multiple alignment of RT sequences and phylogenetic tree with atypically long branches. Using CDD-search fixed the problem and produced a single predicted RVT_1 domain in each of these sequences. Therefore, we used predicted RVT_1 domains based on CDD-searches for these proteins for downstream analyses including multiple alignment and phylogenetic reconstruction.

We used Muscle (v3.8.31) (49) to align the extracted RVT_1 domains in the RT sequences, and then used FastTree2 (50) to build a phylogenetic tree of all the RT sequences, using bootstrap value of 100. By examining the phylogenetic tree, in combination with genomic context analysis, we confirmed the grouping of the RT sequences in the different classes, and for a small number of cases, re-assigned their classes (see Results). We also added new classes. In total, all RTs can be grouped into 41 classes.

Extracted RVT_1 domains for each class were aligned separately using Muscle, and after re-formatting the alignments from fasta to stockholm, we used hmmbuild (51) to obtain hmm models for each class. Then, all of these hmm models (for different classes) were combined into one model (RVT-All.hmm).

### MyRT for identification and characterization of RTs in genomes and metagenomes

**Figure 1.**
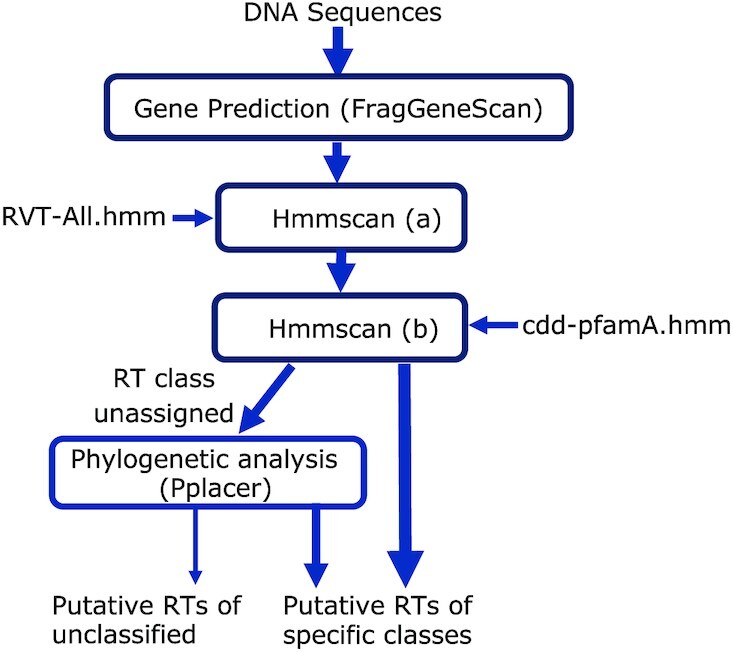
Flowchart of prediction of RTs and their classes. Most RTs can be assigned to specific classes using similarity searches (hmmscan), and a small number of RTs can be assigned to a specific class by combining phylogenetic analysis.

We developed myRT for identification of RTs in genomes and metagenomes. MyRT is based on similarity search against the class-specific RT HMMs, facilitated with phylogenetic analysis by pplacer (52) for the cases when no clear classes can be inferred based on similarity search. First, FragGeneScan (version 1.31) (53) is used to quickly predict the protein coding genes in the input genome (or metagenome); however, if prediction of protein coding genes (given in a gff file) is available, protein sequences will be generated based on the input gff file instead. Next, our pipeline uses predicted protein sequences to find all the RVT_1 domains encoded by the input genome in two steps (see Figure 1):

- Identification of initial putative RT proteins using a focused search of RT domains in all proteins. In this step, hmmscan is used to search all predicted proteins against RVT-All.hmm we created that contains only HMMs of class-specific RTs, with e-value of 10^−3^ (-E 0.0001 –domE 0.0001) as the threshold. Proteins that are predicted to contain RT domains are considered candidates. Since RVT-All.hmm only contains RT domains, some of the identified candidates are likely to be false positives and need to be filtered out by the following step.
- Refinement of RT protein candidates by expanded search of domains in the RT candidates. In this step, hmmscan is applied to search candidate RT proteins against HMMs of a large collection of domains (cdd-pfamA.hmm, which contains a total of 59 083 CDD and Pfam-A domains). Candidates that don’t contain a RT related domain (i.e. RVT_1, group_II_RT_mat, RT_G2_intron, etc.) are considered false positives and are excluded from further analysis. This step is crucial for filtering the false positives (e.g. genes containing DNA binding domains), and the combination of this step with the previous step provides a fast prediction with a high precision and recall.

Classification of putative RT proteins is based on the above hmmscan search results, and in some cases an extra step of phylogenetic placement of the proteins in the tree of RTs. Given a putative RT protein, we consider that its RT class can be confidently assigned, if only one class of RT domain is found by hmmscan, or the top hit has a significantly lower *E*-value than the rest (i.e. *E*-value of the second hit is 10^5^ folds higher than the *E*-value of the top hit). In the cases when a class cannot be assigned, myRT keeps the top three hits, and relies on an extra step based on the placement of the putative RT protein in the phylogenetic tree of known RTs for final assignment of the class for the putative RT protein. The phylogenetic tree of known RTs was inferred by FastTree2 (with a bootstrap value of 100) using multiple alignment of RVT_1 domain sequences as the input. The reference tree (compatible with pplacer) was compiled using Taxtastic. To place a putative RT protein in the phylogenetic tree, first hmmalign is used to combine the putative RT with the reference hmm model. Then pplacer (52) is used to place the query sequence on the tree, and Treeio (v1.10.0) (54) and castor (55) R packages are used to parse the pplacer result. If the putative RT is placed on a leaf node, then the putative RT is assigned the class of the leaf node; otherwise if at least 90% of the leaf nodes in the subtree rooted at the putative RT share the same class, this class will be assigned to the putative RT. The confidence of the prediction at this step will be determined based on like_weight_ratio reported by pplacer, unless pplacer suggests several placements with similar like_weight_ratio, where the difference between second like_weight_ratio and first one is <0.25, in which we will report the result with a confidence value of 0. Finally, if the predicted class based on phylogenetic placement is consistent with hmmscan results (i.e. the prediction is among the top three hmmscan hits), this class will be selected as the final predicted class for the putative RT; otherwise, myRT reports all possible classes. We chose the parameters empirically.

### Genomic neighborhood analysis for putative RTs

To provide genomic neighborhood information of putative RTs, myRT examines up to four neighboring genes for each putative RT (up to two genes downstream, and up to two genes upstream of the RT, with a maximum intergenic region of 2 kbps). The neighboring genes together with the putative RT proteins will be searched against cdd-pfamA.hmm (using hmmscan and a maximum *E*-value of 10^−3^) to annotate the proteins encoded by these genes. The results can be used to infer domains that are frequently fused to the RVT_1 domains of the putative RT proteins, and the frequent domains encoded by the neighboring genes of the RT gene. We note when the predicted class of RT is CRISPR, but the putative RT gene has no nearby *cas* genes (e.g. *cas*1 and *cas*2), myRT will re-assign the class as CRISPR-like. The web version of myRT provides visualization of the prediction of putative RTs along with their genomic neighborhood.

MyRT website also provides statistical analysis of the domains found in RTs and their genomic neighborhood, and allows domain search in the genomic neighborhood of RTs. For each RT class, we compiled a list of domains that co-occur with this specific class of RT along with the frequency of the co-occurrence in complete and draft genomes. Some domains are ubiquitous while others tend to be associated with a certain type of RT. To quantify the specific association of some domains with the different types of RT genes, we proposed a *specificity score* of a domain for a particular class of RT, which is the number of genomes containing this domain in the neighborhood of the particular class of RT divided by the number of times that the domain is found in the neighborhood of any of the RTs. The specificity score ranges between 0 and 1, with 1 indicating that a domain is exclusively associated with a particular RT class. Using these tools, we can see that 46% of the UG28b RTs co-occur with VirE_N domains (see Supplementary Figure S1 for the report), and although only 7% of Retron-RTs found in complete genomes co-occur with PRK10473 (MdtL family multidrug efflux MFS transporter), among the RTs that co-occur with this domain (including 207 Retrons-RT, 2 GII RTs and 1 UG7-RT), 99% of them were Retron RT (i.e. PRK10473 is highly specific to Retron RT with a specificity score of 0.99; see Supplementary Figure S2).

### Genomes and metagenomes

We applied myRT to predict putative RTs in reference genomes (including complete and incomplete) and selected metagnenomes. Reference genomes were downloaded from the NCBI ftp website as of 22 October 2020. For complete genomes, we used NCBI’s prediction of putative coding genes, whereas for draft genomes, we used FragGeneScan (53) to predict protein coding genes. For metagenomes, the reads were trimmed using Trimmomatic (56), and paired reads were assembled using MetaSPAdes (53). FragGeneScan was then used to predict putative coding genes from the assemblies of the metagenomes.

## RESULTS

### Reclassification of some RTs, expansion of rare RT classes and addition of new classes

We improved the collection of RT sequences and their models from three different aspects: reclassifying some RT sequences that were likely misclassified; adding more sequences for rare RT classes for model construction, and adding new RT classes.

We first applied the class-specific HMM models to predict and assign classes to the sequences in the initial training dataset. More than 99% of predictions agreed with the old classification. The rest could be either errors in the old classifications or misclassifications introduced by myRT. We analyzed these cases further, combining their sequential, genomic neighborhood, and phylogenetic information. Further we used CRISPRone (57), myDGR (5), and groupii (39,40) to confirm RTs involved in CRISPR-cas, DGR and group II introns, respectively. We excluded three sequences that don’t contain RVT_1 domain, including YP_002455118.1 (WP_000385107.1), KQB14190.1 and WP_009625650.1. In addition, we were able to revise the classification for a total of 10 RTs summarized in Table 1 and Supplementary Table S1. For example, EGP13976.1 is one of the 155 DGR RTs identified by (42), but it is the only one (out of 155) that is not part of a DGR system; we regrouped it as an intron-RT with appended GIIM domain.

**Table 1.** Re-classification of 10 of the previously labeled RTs

Accession number	Old classification	New classification	Co-occurring domain(s)
EGP13976.1	DGRs	Group II introns	GII-RT
YP_001397265.1 (EDK35894.1)^a^	DGRs	Group II introns	Intron_maturas2^c^
AFZ16538.1 (WP_015180701.1)^a^	UNC	AbiA	MazF, HTH_XRE
NP_442332.1^a^	UG3	UG7	HicB
AFY59940.1^a^	UG3	UG7	gluta_reduc_2
AGA07305.1^a^	UG6	UG12	YjgR
EPZ72367.1^a^	UNC	UG15	GepA
AGI67543.1^b^	Group II like 3	UG3	UG8
AEJ99900.1^b^	Group II like 4	UG4	FimD, FimA
CCF10237.1 (EQR96236)^b^	UG11	Retrons	Spo0J

^a^ These RTs are from (4).

^b^ These RTs are from (6).

^c^ These domains are encoded by the same gene that encodes the RT domain.

**Table 2.** Evaluation of myRT on the Simon 2019 collection of retron RTs (16), all of which were predicted as retron RT by myRT

Known retron	Genome	RT coordinates	Identity%$^\&$
EC48$^\#$	*Escherichia Coli* DE147	LFQP01000005.1_154506_155696_-	50
EC67$^\#$	*Escherichia coli* S10	CP010229.1_4712073_4713833_-	61
EC73$^\#$	*Escherichia coli* M10	CP010200.1_2393178_2394128_+	36
Ec78$^\#$	*Escherichia coli* 102598	JHRW01000018.1_27622_28557_+	48
EC83$^\#$	*Escherichia coli* 05-2753	CXYK01000012.1_74586_75524_+	47
Mx65	*Myxococcus xanthus* DSM 16526	FNOH01000027.1_37959_39242_+	53
Eco8$^\#$	*Escherichia coli* 200499	CYGJ01000003.1_369367_370491_+	47
Se72	Salmonella enterica*	AMMS01000284.1_2640_3671_-	48
Vc137$^\#$	*Vibrio cholerae* 2012EL-1759	JNEW01000012.1_609188_610135_+	49
Vp96	*Vibrio parahaemolyticus* S119	AWJG01000250.1_32_1054_+	49
YF79	*Yersinia frederiksenii* ATCC 33641	KN150731.1_1692670_1693602_-	50

$^{\#}$ These retrons function as anti-phage defense systems. *Salmonella enterica enterica sv. Heidelberg 579083-10. $^{\&}$ This column lists the highest sequence identity between the predicted RT and the RVT_1 domains used to build the HMMs for the different classes of RTs.

**Figure 2.**
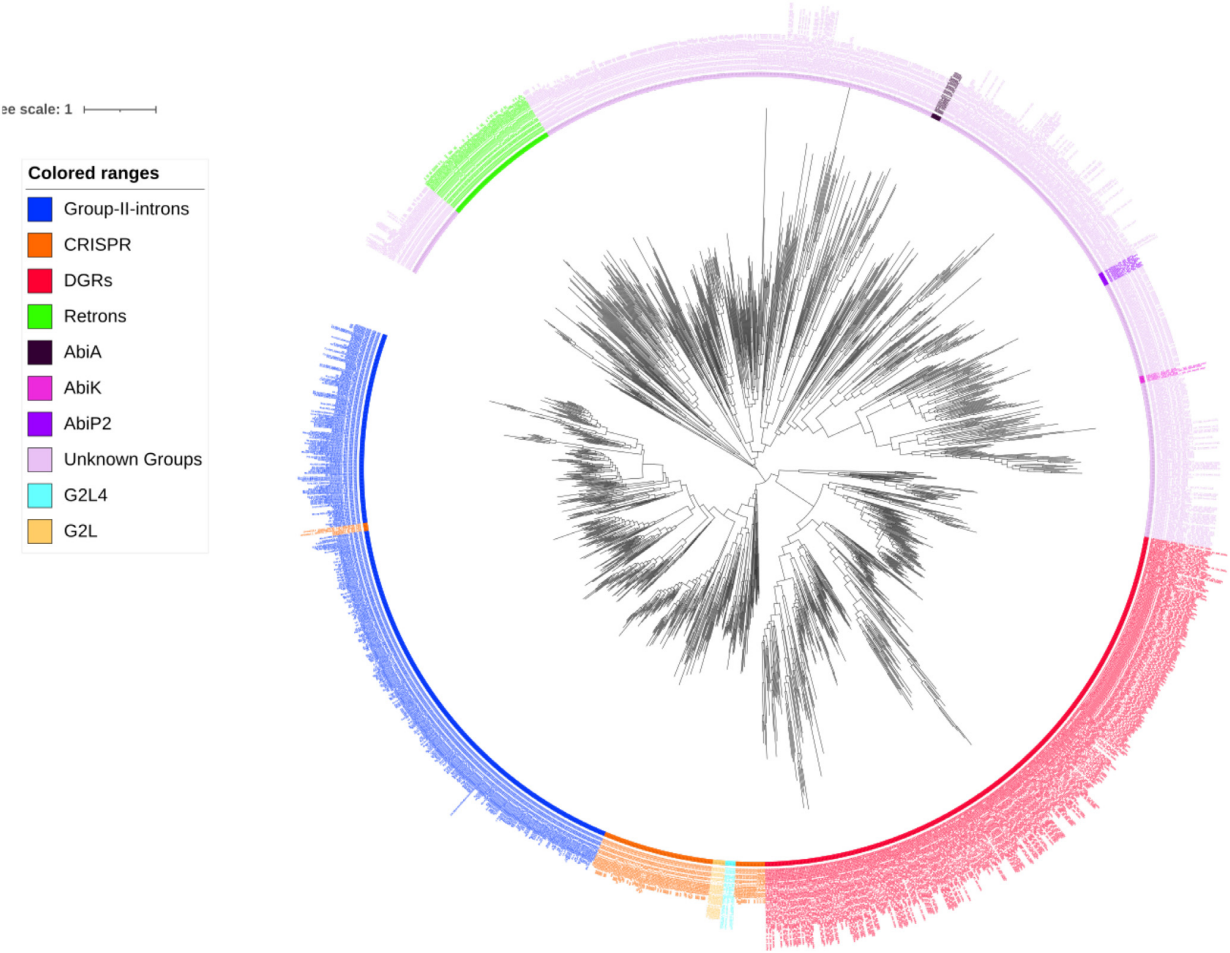
Phylogenetic tree of different RTs. The tree was inferred by FastTree2 (50) using the multiple alignment of RVT_1 domains as the input. The tree was visualized using iTOL (58), and the interactive version of the tree is available at iTOL-myRT (https://itol.embl.de/tree/6624481121182221623936089).

We improved the hmm models for the classes with few representatives. We applied myRT to find putative RTs in 20 036 complete and 118 883 draft prokaryotic genomes, and extracted new RTs that belong to classes with few representatives (including UG15 and UG21). Then, we verified the accuracy of these new classifications by adding the RVT_1 motif sequence of these putative RTs to the phylogenetic tree of RTs (see Figure 2), to make sure that they fall in the right clade. After adding these newly classified RTs to the training data, we rebuilt the hmm models for these rare classes. For instance, starting from six UG25 RTs, we were able to include an additional of 19 sequences and used a total of 25 sequences to build the HMM of UG25.

**Figure 3.**
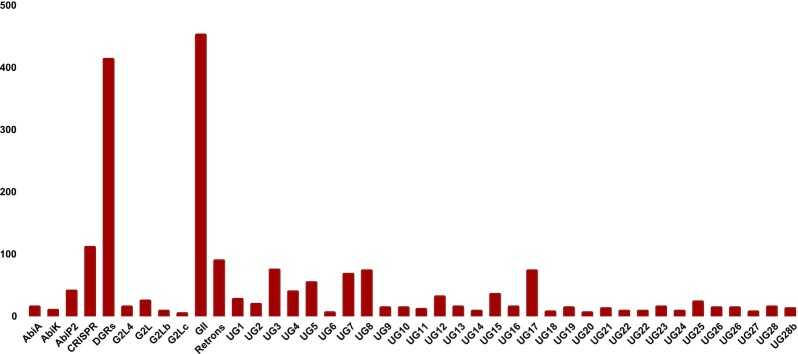
Barplots of the number of RTs in each class in our final training set.

We added new classes of RTs based on a combination of sequential, phylogenetic tree and genomic neighborhood analyses. We divided G2L RTs into three classes (G2L, G2Lb, G2Lc) based on their placement on the phylogenetic tree and how these sequences are clustered together in a branch in the phylogenetic tree (see Figure 2). Similarly we divided UG28 RTs into two classes and called the smaller class UG28b. The hmm of UG28b was built using 15 sequences that form a separate clade in the phylogenetic tree. In addition, UG28 and UG28b RTs have different domains in their genomic neighborhood—45% of UG28b RTs co-occur with VirE_N domains. The final RVT-All.hmm contains 45 HMM models (for 41 RT classes; some classes have more than one model, and CRISPR and CRISPR-like share the same HMM models) built from a total of 1988 RVT_1 sequences. Number of representatives of each class, and multiple alignment of RVT_1 motif sequences of each class are available at myRT website. See Figure 3 for the size distribution of the different RT classes.

### Evaluation of myRT using three independent collections of RTs

We applied myRT to three independent collections of RTs for evaluation. All results showed that myRT gave accurate classification of RTs. Supplementary Tables S2– S4 provide access of myRT results for these three collections.

The first collection contains CRISPR-Cas reverse transcriptases (branch 1 - branch 10) from (36) that were not used in building the hmm models. The last column of Supplementary Table S2 shows the identities of each RT to the RVT_1 motif sequences used in building RVT-All.hmm. KKO19091.1 (LAQJ01000220.1_7006_7917_-) despite being a CRISPR-RT shares 39% sequence identity with RVT_1 domain of AGB41082.1 (GII RT), and shares 37% sequence identity with WP_012599795.1 (CRISPR-Cas RT), and was correctly predicted as CRISPR-Cas RT by our pipeline solely based on hmmscan results. GAN31766.1 (RT#1 in BAFN01000001.1) shares 71% identity with CAJ74578.1, and based on CRISPRone results seems to be adjacent to a *cas4* gene, whereas CAJ74578.1 which is adjacent to a *cas1* gene. Our pipeline did not recognize the *cas*4 gene, only reported a gene encoding GxxExxY domain, and thus labeled this RT as CRISPR-like. The last three RTs in Supplementary Table S2 are associated with type I-E CRISPR-systems, have an adjacent *cas*3 gene, and two of them have an adjacent gene encoding AbiEii domain (atypical for CRISPR-Cas RTs). Although we do not have specific models for these unusual CRISPR-Cas RTs, LAKD01000050.1 was correctly predicted as a CRISPR RT by myRT. MyRT predicted AJKO01000007.1 as a UG2 RT instead of a CRISPR RT, and we believe the original annotation in (36) was wrong: the RT gene was found in *Streptococcus oralis* SK10 (EIC80228.1), which encodes for a type I CRISPR-Cas system, but the *cas* genes and the RT gene don’t co-locate; and the RT was also annotated as a UG2 RT in a later study (35). MyRT couldn’t assign the last two putative RTs (DS570667.1 and CP007699.1) to a specific RT class so annotated them as unclassified (UNC) RT. Overall, we estimate that myRT can predict a CRISPR RT as CRISPR/CRISPR-like RT with an accuracy of 93%.

We then tested our model on datasets from (6) (excluding unclassified RTs). To fairly assess myRT’s performance we excluded RTs that shared more than 87% identity with our RT collection. The majority of the retained ones share 30%-50% identity with our training set which is normal considering that all of them have conserved RT domains. The test set used for this evaluation, and the results can be found in Supplementary Table S3. Most of the predictions matched with the groupings from this collection, except two putative G2L5 (GII-like-5) RTs, ZP_01854760.1 and ZP_01851752.1, from *Gimesia maris* DSM 8797 which were predicted as G2Lb, and CRISPR-like by myRT, resulting in 90% accuracy overall. We note the two putative G2L5 RTs share low sequence identity (28%) with each other, and both seem to be located in transposons: ZP_01854760.1 shares 33% sequence identity with YP_552148.1 (a GII RT), but lacks the GIIM domain; and the other one ZP_01851753.1 has HTH_Tnp_1 (helix-turn-helix) and Tra5 (transposase InsO and inactivated derivatives) encoded by its flanking genes.

Finally, we tested our pipeline on a dataset of 16 retrons with validated RTs (16), 12 of which were recently experimentally proved to function as anti-phage defense systems. Five of these RTs were already in our training set, yet the other 11 shared less than 61% sequence identity with the RTs included in our training data. MyRT was able to correctly predict all of them as retron RTs, providing an accuracy of 100% (see Table 2 and Supplementary Table S4). One example is the Ec48 retron system, which has proved vigorous defense against *Siphoviridae*, *Myoviridae* and *Podoviridae* phages. We note in the genomic neighbhorhood of the Ec49 retron system, there are genes encoding for an AbiP2 RT (sharing 99% identity with CAJ43157.1, AbiP2 RT of *Enterobacteria* phage P2-EC58), and other domains include Q (portal vertex) and phage_GPA (bacteriophage replication gene A protein).

### Putative RTs identified in bacterial genomes

**Figure 4.**
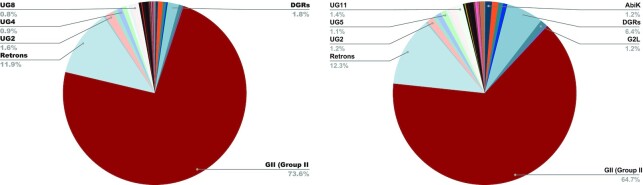
Distribution of different classes of RT in complete (left) and draft (right) bacterial genomes.

We applied myRT to predict putative reverse transcriptase, alongside their class in all complete and draft bacterial genomes. In total, 8,244 out of 20,036 complete genomes, and 118,841 out of 262,497 draft genomes each contain at least one putative RT. This collection is easily accessible through our web-server. We note that for genomes with predicted RTs associated with DGRs or CRISPRs, DGR prediction (by myDGR) and CRISPR-Cas prediction (by CRISPRone) are also provided. Figure 4 shows the distribution of each RT class in complete and draft bacterial genomes. Just as expected, group II intron is the most prevalent class of RT (≈65%), followed by retron (≈12%). Retrons were recently found to provide phage defense mechanisms, and myRT predicted a total of 2973 and 30 825 retron RTs in the complete and draft genomes, respectively (see Supplementary Table S5). These putative retron RTs will be useful for further study of the function and distribution of the retron RTs in bacterial genomes. Below we show several cases of myRT predictions for demonstration purposes. MyRT results for these reference genomes and plasmids are available in Supplementary Table S6.

**Figure 5.**
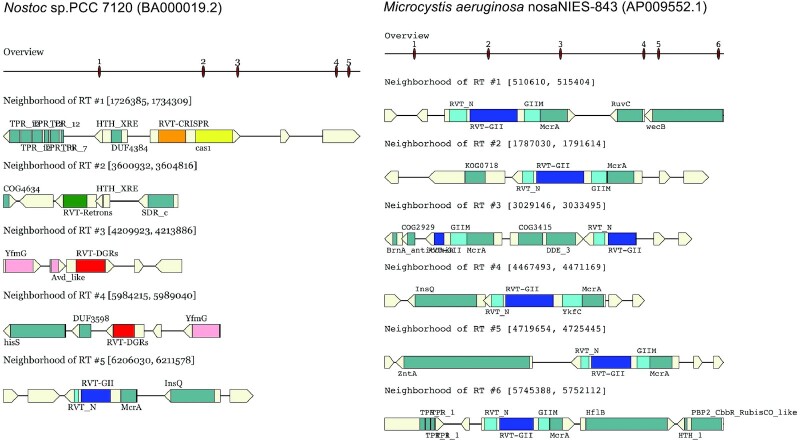
MyRT prediction results for *Nostoc sp*. PCC 7120 (left) and *Microcystis aeruginosa* NIES-843 (right). The ‘overview’ view shows the locations of the predicted RT genes along the genome, and the zoom in views below each show one RT gene and its neighborhood. Arrows represent the genes, with the different regions encoding different domains in colored rectangles. All six RTs in *Microcystis aeruginosa* are GII intron RTs (in blue), whereas *Nostoc* sp. contains RTs of different classes (in different colors). The RVT_1 domains in the RTs are specified with the RT class (so these domains are named as RVT-CRISPR, RVT-DGR, RVT-GII, etc. in the plots).

Figure 5 shows myRT predictions of two genomes. The first example is *Nostoc* sp. PCC 7120 and its plasmids. The two plasmids each contain a gene encoding group-II intron RT, and the genome encodes four classes of RT: DGR, CRISPR, retron and group II intron as shown in Figure 5 (left, only showing predicted RTs in the genome; see predicted RTs in the genome and the plasmids at myRT website). The second example is *Microcystis aeruginosa* NIES-843, which has seven RTs all related to group II introns. Six of the seven RTs are almost identical (sharing 97–99% identity), and they only share low identity (51%) with the seventh RT (which shares 65% identity with ACV02121.1, a group II intron RT in *Cyanothece* sp. PCC 8802). As seen in Figure 5 (right), six of these group II intron RTs have a fused McrA domain (5-methylcytosine-specific restriction endonuclease McrA).

Surprisingly, we observed that *Bacillus thuringiensis* YBT-1518 and its plasmids have a large number of group II intron RTs (74 GII RTs), and one RT with unknown function, which is similar to class of UG4 RTs, but doesn’t have Fimbrial domain in its genomic neighborhood. Out of these 74 RTs, 60 of them are identical to AHA69388.1 (RT #1) and 3 of them are identical to AHA69975.1 (RT #6). AHA69975.1 is adjacent to a potential virulence gene with VirD4 (Type IV secretory pathway component) domain.

Application of myRT to the complete bacterial genomes resulted in the identification of 29 AbiA RTs. More than 70% of these AbiA RTs have a fused HEPN_AbiA_CTD domain. Examples of AbiA and AbiK, can be found in U17233.3 (*Lactococcus lactis* plasmid pTR2030), and in U35629.2 (*Lactococcus lactis* plasmid pSRQ800), respectively. Based on the genomic neighborhood, this AbiK seems to be part of a restriction-modification system. The third class of Abi RTs is AbiP2, an example of AbiP2 is found in *E. coli* 536. Some AbiP2 RTs are located in a CRISPR-Cas loci, and may be associated with CRISPR-Cas systems as discussed earlier. Other examples of Abi RTs can be accessed through myRT-collection.

When applied myRT to predict putative RTs in complete genomes, about 93% of the putative RT had their class assigned solely based on hmmscan results. For the rest, phylogenetic information was used to assign a class to 85% of them. Among 25,570 predicted RTs in complete genomes, 88 RTs (less than 1%) remain unclassified, 46 of which share more than 56% identity with YP_003455357.1 (CBJ12261.1) unclassified RT in *Legionella longbeachae* NSW150. Table 3 and Supplementary Table S7 contains some of the examples where phylogenetic information helped improve the classification of putative RTs. For instance ADE85032.1 was predicted as a CRISPR RT. Had we only used hmm models, it would be predicted as GII RT. This RT has a fused Cas1 domain, and its encoding gene has a *cas6* gene in the genomic neighborhood which indicates this RT is indeed related to a CRISPR-Cas system. The other set of examples include 10 UG3 RTs that otherwise would be classified as Unclassified RTs (e.g. UG3/DGRs/UG17), and we note that nine of these predicted UG3 have UG8 in their neighborhood (UG3 and UG8 tend to co-occur according to our training data and previous studies (6,59)).

**Table 3.** Potential improvements of RT classification by using phylogenetic information

Accession number	Gene coordinates	hmmscan	pplacer	Genomic neighborhood
-	KB901875.1_2019009_2019602_+	CRISPR/**GII**	GII	GIIM
ANU66363.2	CP015403.2_1765974_1766285_-	DGRs/**GII**	GII	GIIM
ABW11582.1	CP000820.1_2576306_2576710_+	**GII**/CRISPR	GII	GIIM
ACN15726.1	CP001087.1_3000453_3001124_-	DGRs/**GII**	GII	GII
BAZ36932.1	AP018280.1_22270_22950_+	DGRs/**GII**	GII	McrA
ABW09889.1	CP000820.1_498987_499841_+	DGRs/**GII**	GII	INT_RitC_C_like
BBI33370.1	AP019400.1_3194415_3197627_+	DGRs/**UG6**/UG23	UG6	nitrilase
AIG26831.1	CP007806.1_2784436_2786124_-	UG15/**AbiK**/UG12	AbiK	HTH_21, InsE
ARW20833.1	CP021477.1_3770_5002_-	Retrons/**AbiA**/UG9	AbiA	SLATT_5
AQT81505.1	CP019882.1_4836808_4838490_+	UG9/**UG23**/UG19	UG23	HTH_17
AHX61471.1	CP007567.1_2408799_2408972_+	**Retrons**/UG24/DGRs	Retrons	zf-IS66
AUJ27137.1	CP015444.1_135192_137216_+	UG12/**AbiK**/UG15	AbiK	KAP_NTPase
AUI76834.1	CP015498.1_1856458_1858044_-	UG12/**AbiK**/UG15	AbiK	Abi_2, GlpR
AZA22328.1	CP031016.1_1912675_1914699_+	UG12/**AbiK**/UG15	AbiK	AbiH

The ‘Gene coordinates’ column lists the coordinates of the predicted RT gene in the corresponding genome. The ‘Initial prediction’ and the ‘Final prediction’ columns list the predicted class for each putative RT before and after using the phylogenetic information, respectively. The ‘Neighborhood’ column shows the adjacent domain that are found together with the predicted RT in the genomic sequence.

### Application of myRT to predict RTs in metagenomes

**Figure 6.**
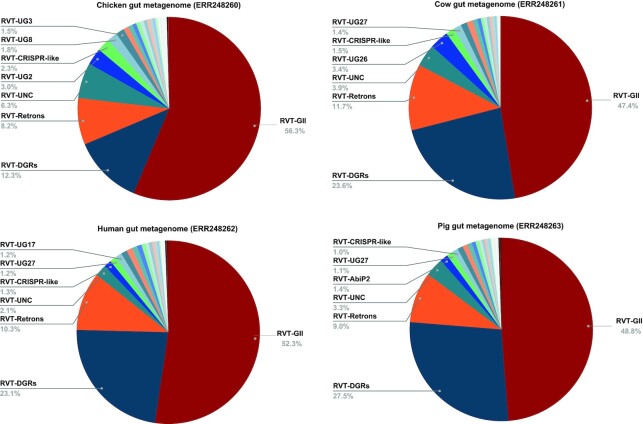
Distribution of RT classes in representative gut metagenomes of human, chicken, cow, and pig.

For demonstration purposes, we applied our pipeline to identify putative RTs in metagenomes. The first set contains four gut metagenomes (ERR248260-ERR248263) from fecal microbiota of human, chicken, cow, and pig from (60). As seen in Figure 6, all gut metagenomes contain higher proportions of DGR-related RTs as compared to the reference bacterial genomes (see Figure 4), and the pig gut metagenome has the highest proportion of DGR RTs among all. The pig gut metagenome contains 1350 putative RTs, including 659 GII RTs, 380 DGR RTs (348 after removing sequence redundancy by cd-hit (46) using sequence identify cutoff of 70%), 119 retron RTs and other classes of RTs. Out of 348 non-redundant DGR RTs in this dataset, 157 share less than 70% identity with the DGR-RTs from complete and draft bacterial genomes which contains 4465 non-redundant DGR-RTs (cut-off value: 0.7). Using myDGR, we were able to identify 38, 15, 15 and 15 complete DGRs (a typical DGR system contains a RT gene, a template region TR, and a target gene containing the corresponding variable region VR) in the human, chicken, cow and pig gut metagenome, respectively, reflecting the fragmented nature of the metagenome assemblies (many of the contigs are very short). Supplementary Table S8 includes the links to myRT and myDGR predictions of these metagenomes.

To further investigate if pig gut metagenomes generally have a high proportion of DGR-RTs, we tested four additional pig gut metagenomes (ERR1135178–ERR1135181) from (61). According to myRT results, even higher proportions of DGR-RTs (42–49%) were observed in these pig gut metagenomes (see Supplementary Table S8 and Supplementary Figure S3).

### Genomic context preferences of different classes of RTs

With predictions of putative RTs in complete genomes, we were able to identify domains that are frequently found in the proteins encoded by the neighboring genes of putative RTs (including those that are fused with the RT genes). Table 4 lists some of the co-occurring proteins/domains found in complete genomes (Supplementary Table S9 shows the frequent domains observed in the genomic neighborhood of RTs used in our RT collection, i.e., training data). Among non-redundant (identity $90\%$) putative RTs in complete genomes, 87% of CRISPR-Cas RTs are found to co-occur with Cas1 domain, and 64% co-occur with Cas2. 62% of DGR-RTs are found to co-occur with Avd_like domain; Avd_like is found in bacterial accessory variability determinant (bAvd) proteins) in DGR systems. 82% of UG17 RTs are found together with SLATT_5 domain (families of SLATT domains are predicted to be associated with cell-suicide and diversity generating (62)). About 97% UG3 RTs are adjacent to a UG8 RT, and 90% of UG8-RTs are adjacent to a UG3 RT. We note some domains are fused with the RT domains (RVT) in the same proteins. Table 4 shows three fusion instances (e.g. group_II_RT_mat is found to be fused with UG6 RT in 93% of the instances that contain the RT in complete genomes).

**Table 4.** Frequent domains encoded by the genes that are in the neighborhood of or fused to the genes encoding RTs in complete genomes

RT class	Domain	Co-occurrence frequency
CRISPR	Cas1 (Cas1_I-II-III,cas1,Cas_Cas1,cas1_HMARI,cas1_CYANO)	87%
CRISPR	Cas2	64%
DGRs	Avd_like	62%
GII	GIIM (fused)	58%
UG3	UG8	97%
UG4	Fimbrial domain (FimA, PRK15287, FimD, FimC, PRK15288)	66%
UG5	nitrilase	50%
UG6	group_II_RT_mat (fused)	93%
UG8	UG3	90%
UG9	PRK14975	94%
UG17	SLATT_5	82%
UG10	AE_Prim_S_like/COG4951	61%

Other domains encoded by the genes that are occasionally found in the neighborhood of RT genes include RelB (PF04221; antitoxin), RelB_dinJ (antitoxin), dinJ-yafQ (toxin-antitoxin module), HTH_XRE (Helix-turn-helix XRE-family), HTH_Tnp_1, Trypsin_2, AbiEii (Nucleotidyl transferase AbiEii toxin, Type IV TA system), DDE_Tnp_1 (transposase), metallo-hydrolase-like_MBL-fold, mazF, xerC, AcrR (DNA-binding transcriptional regulator), AAA (ATPase family), SMC_prok_B, dnaG, DNA_pol_A, Phage_integrase, InsE (Transposase and inactivated derivatives), T_den_put_tspse (putative transposase), and RAYT (REP element-mobilizing transposase), etc.

We observed nine reference genomes that have plasmids encoding group II introns RTs, and their adjacent genes are *bla*_*IMP*26_ multidrug resistance genes, which encode proteins containing IMP_DIM-like_MBL-B1 domain (cd16301). We expanded our analysis and compiled a list of 25 plasmids that carry IMP resistance genes and have a group II intron RT (see Supplementary Table S10). All of these group II intron RTs, have IMP_DIM-like_MBL-B1 in their genomic neighborhood except one (KX711880.1), and some also have Multi_Drug_Res (pfam PF00893) domain in their flanking genes. It seems that all of these group II intron RTs are almost identical to Kl.pn.I3 (ACJ76645.1). This result suggests the association of intron RTs and the multidrug resistance.

**Figure 7.**
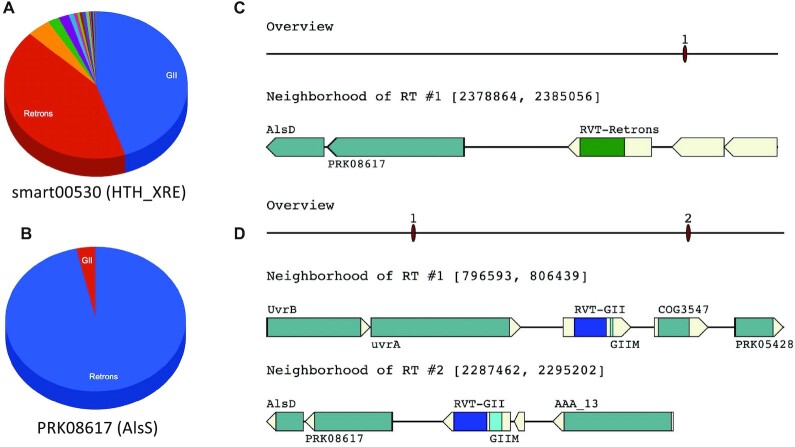
Representative protein domains that are associated with RT genes of different types. (**A**) and (**B**) are piecharts representing the distribution of domain smart00530 (helix-turn-helix XRE-family like proteins ) and PRK08617 (AlsS) co-occurring with different RTs, respectively; and (**C**) and (**D**) show the association of AlsS with Retron RT (in *S. aureus* subsp. aureus JH9, highlighted in green in the figure), and GII intron (in *S. aureus* subsp. aureus JKD6159, highlighted in blue), respectively. Note there are two GII RT genes in *S. aureus*. aureus JKD6159, but only one (in locus 2) has *AlsS* gene in the neighborhood. A gene encoding for AlsD (Alpha-acetolactate decarboxylase) is also found in the neighborhood of GII RT, adjacent to PRK08617 (AlsS). All plots in this figure were automatically generated by myRT.

A recent work studied the domains associated with Retron RTs, and ATPase (COG3950) was one of the domains found in the genomic neighborhood of Retron RT genes (clade 1 in (63)). Our analysis also revealed this association with a specificity score of 0.96 and 1 in complete and draft genomes, respectively. The high specificity score for this domain from our analysis provides a quantitative metric showing the specific association of this domain with Retron RTs (but rarely with RTs of other classes). Our results also showed several other domains that are (almost) exclusively associated with Retron RTs, including PRK10473 (MdtL family multidrug efflux MFS transporter) and PRK08617 (acetolactate synthase, AlsS); by contrast, smart00530 is frequently found in the Retron RT gene neighborhood, however, it is also frequently found together with group II RT (GII) and other types (see Figure 7A and B for a comparison). PRK08617 was found in the neighborhood of Retron RT genes in many *S. aureus* genomes (including 110 complete and 3319 draft genomes; see Figure 7C for an example, and more details at myRT website), and is also associated with GII RT but only in four genomes, including one *S. aureus* genome (see Figure 7D), *Bacillus thuringiensis* YBT-1518, and two genomes of *L. reuteri*. *S. aureus* AlsS was reported to confer resistance to nitrosative stress and contribute to the successful infection of murine macrophages, and resistance of *S. aureus* to beta-lactam antibiotics (64). Our genomic context analysis revealed a strong association of AlsS (and AlsD) with Retron RTs, especially in *S. aureus*, suggesting a possible connection of Retron RTs with these biological processes in *S. aureus*.

## DISCUSSION

In this study, we provided a tool for prediction, and classification of reverse transcriptase (RT) in bacterial genomes. Reverse transcriptases, the enzymes that convert RNA into cDNA, play substantial roles in different systems such as Diversity Generating Retroelements (DGRs), group II introns, CRISPR-Cas systems, retrons, etc. Identification of these RTs can provide us with information about the underlying interactions between phage and bacteria, archaea and archaeal viruses, and so forth. It can also help us to determine the origin of the RT, does it come from another species, or is it native (for instance, DGR RTs that come from phage)? Classification of RTs can also be extremely helpful when it comes to biotechnological/medical applications of certain classes of RTs, such as utilizing group II introns RTs as targetron and thermotargetron, and make use of retrons RTs in CRISPEY, SCRIBE and HiSCRIBE methods, or as anti-phage defense systems. As RTs from each class have similar functions, characterization of every single RT is of importance, as it can shed light into identification of other RTs from the same class/family. Experimental studies can come to rescue, and identify the function of less-known/unknown groups of RTs. Thus, we provide a list of RTs in every single class (known/unknown), and even unclassified RTs in complete and bacterial genomes, as we believe these lists can be used by researchers in computational and experimental fields.

We showed that myRT provides accurate predictions for most RT classes, esp. the large classes such as GII, retron and DGR RTs. However, we acknowledge that smaller or unknown classes of RTs will be more challenging to predict, because either the HMM models used for prediction and classification were built from a smaller number of sequences, or because some classes are very similar to each other and are close in the phylogenetic tree of RTs. Nevertheless, myRT would be able to predict those RTs, which can be further analyzed by using other information.

We expect that genomic neighborhood information can help provide insights into the putative function of unknown classes of RTs, including UG1-UG28, and other classes of RTs. Fused domains in these RTs, alongside the information about the domains in the flanking genes of RTs in each class, can provide us with some insights into the functions of these RTs. Also, as we collect more data for each class, this information can be used or examined by researchers. For example, SLATT_5 is frequently seen next to UG17 RTs. Our analyses show that 81% of RTs in complete genomes that have UG17 RT, also have SLATT_5 in the genomic neighborhood of the UG17 RT. Similarly, 76% of UG17 RTs in draft genomes have a SLATT_5 domain in their flanking genes. An example of SLATT domain next to a reverse transcriptase in *Salmonella enterica* subsp. enterica serovar 9,12:l,v:- str. 94293 is mentioned in (62). This reverse transcriptase shares 92% identity with WP_015462025.1, UG17 RTs from *Edwardsiella piscicida* C07-087, which is mistakenly labeled as CRISPR RT in several other articles, yet our phylogenetic analysis showed that this RT groups with UG17 RTs, it has the SLATT_5 domain in its adjacent neighboring gene, and importantly we couldn’t detect any Cas neighbors, or CRISPR systems in this reference genome.

## DATA AVAILABILITY

MyRT is available to be used stand-alone (https://github.com/mgtools/myRT), and online (https://omics.informatics.indiana.edu/myRT/). Predictions of RTs in reference genomes and selected metagenomes are available at https://omics.informatics.indiana.edu/myRT/collection.php.

## Supplementary Material

gkab1207_Supplemental_File
