# Supplementary material for: Identification and classification of reverse transcriptases in bacterial genomes and metagenomes
Source: Nucleic Acids Res. 2021 Dec 14;50(5):e29. doi: 10.1093/nar/gkab1207 (PMC8934634; doi:10.1093/nar/gkab1207)
Supplement: gkab1207_Supplemental_File [file gkab1207_supplemental_file.pdf]

Supplementary materials: Identification and Characterization of Reverse Transcriptases in Bacterial Genomes and Metagenomes

Fatemeh Sharifi[1] and Yuzhen Ye[2,*]

[1,2] *Luddy School of Informatics, Computing, and Engineering , Indiana University, Bloomington, IN 47408, USA*

[*] *Corresponding author: Yuzhen Ye, yye@indiana.edu*

Supplementary Table 1: Re-classification of 10 of the previously labeled RTs

Accession number	Old classification	New classification	genomic neighborhood	myRT results
EGP13976.1[a]	DGRs	Group II introns	GII-RT, AckA, MscS	myRT prediction
YP_001397265.1 (EDK35894.1)[a]	DGRs[c]	Group II introns	Intron_maturas2[d], BcsQ, RsmG	myRT prediction
AFZ16538.1 (WP_015180701.1)[a]	UNC	AbiA	MazF (toxin), HTH_XRE, Phd (antitoxin)	myRT prediction
NP_442332.1[a]	UG3	UG7	HicB (antitoxin), carbamoyl-phosphate synthase	myRT prediction
AFY59940.1[a]	UG3	UG7	gluta_reduc_2, MdlB (ABC-type multidrug transport system)	myRT prediction
AGA07305.1[a]	UG6	UG12	YjgR (bacterial ATPase), SIR2_2 (sirtuins related)	myRT prediction
EPZ72367.1[a]	UNC	UG15	GepA(phage-associated protein)	myRT prediction
AGI67543.1[b]	Group II like 3	UG3	UG8 RT, HTH_17	myRT prediction
AEJ99900.1[b]	Group II like 4	UG4	FimD (usher protein), FimC (chaperone PapD), Integrase	myRT prediction
CCF10237.1 (EQR96236)[b]	UG11	Retrons	Phage_pRha, KilAC, ApeA_NTD1, Spo0J	myRT prediction

[a] These RTs are from [1].[b] These labels are based on [2]. [c] These labels are based on [3] [d] These domains are fused to the gene on column 1.

Supplementary Table 2: Evaluation of myRT on the collection of CRISPR-Cas RTs from [4]

Genome	RT coordinates#	myRT prediction	identity%&	myRT results
ASPN01000006.1	ASPN01000006.1_20247_22901_+	CRISPR	34	myRT prediction
AQRP01000065.1	AQRP01000065.1_13318_16137_+	CRISPR	34	myRT prediction
JXXW01000010.1	JXXW01000010.1_71385_73319_+	CRISPR	59	myRT prediction
ASAJ01000015.1	ASAJ01000015.1_125843_128860_-	CRISPR	56	myRT prediction
KL370780.1	KL370780.1_93557_95149_+	CRISPR	35	myRT prediction
JWIO01000025.1	JWIO01000025.1_10124_11221_+	CRISPR	38	myRT prediction
BCQS01000026.1	BCQS01000026.1_4694_5707_-	CRISPR	40	myRT prediction
JH470356.1	JH470356.1_27166_28113_+	CRISPR	77	myRT prediction
LLVU01000015.1	LLVU01000015.1_5740_7752_-	CRISPR	57	myRT prediction
LOAS01000028.1	LOAS01000028.1_37027_38064_-	CRISPR	48	myRT prediction
JYJP01000030.1	JYJP01000030.1_21534_23426_+	CRISPR	74	myRT prediction
JH992891.1	JH992891.1_86001_86924_-	CRISPR	75	myRT prediction
ANNX01000115.1	ANNX01000115.1_14418_15371_-	CRISPR	76	myRT prediction
ALWD01000163.1	ALWD01000163.1_24045_25013_+	CRISPR	84.62	myRT prediction
ANNX01000117.1	ANNX01000117.1_21795_22772_-	CRISPR	86	myRT prediction
JH992901.1	JH992901.1_1016250_1017311_+	CRISPR	81	myRT prediction
JXCA01000005.1	JXCA01000005.1_398870_399850_-	CRISPR	82	myRT prediction
ALVY01000183.1	ALVY01000183.1_27387_28304_+	CRISPR	69	myRT prediction
HE972669.1	HE972669.1_45936_46913_-	CRISPR	72	myRT prediction
ALWB01000016.1	ALWB01000016.1_12151_14244_-	CRISPR	64	myRT prediction
ASMA01000004.1	ASMA01000004.1_21607_22458_+	CRISPR	46	myRT prediction
JQFA01000004.1	JQFA01000004.1_1149670_1151637_-	CRISPR	85	myRT prediction
AJLK01000155.1	AJLK01000155.1_941_2959_-	CRISPR	86	myRT prediction
ASZN01000033.1	ASZN01000033.1_1862_3346_-	CRISPR	52	myRT prediction
LAQJ01000220.1	LAQJ01000220.1_7006_7917_-	CRISPR	37	myRT prediction
KK211136.1	KK211136.1_10156_11052_-	CRISPR	62	myRT prediction
BAFN01000001.1	BAFN01000001.1_294229_295173_+	CRISPR-like	71	myRT prediction
AJKO01000007.1	AJKO01000007.1_124518_125894_-	UG2*	34 (UG2)	myRT prediction
LAKD01000050.1	LAKD01000050.1_46946_48151_-	CRISPR*	25	myRT prediction
DS570667.1	DS570667.1_23830_25101_-	UNC*	29	myRT prediction
CP007699.1	CP007699.1_7617452_7618696_+	UNC*	38 (GII)	myRT prediction

* These 4 RTs were referred as "RTs with unusual RT-associated CRISPR-Cas architectures" in [4]. #: Coordinates of the predicted RT genes are presented in the genome/contig_start_end_strand format, which shows the start, end, and strand of the protein coding regions. & This column lists the highest sequence identity between the predicted RT and the RVT_1 domains used to build the HMMs for the different classes of RTs.

Supplementary Table 3: Result of testing myRT on the datasets from [2]

Accession number	Gene coordinates	Class	myRT Prediction	myRT results
CAA78293.1	AHAX01000006.1_19205_20143_+	Retrons	Retrons	myRT prediction
AAM42896.1	AE008922.1_4324395_4326089_+	Retrons	Retrons	myRT prediction
AFY43443.1	CP003548.1_3308170_3310188_-	CRISPR*	CRISPR	myRT prediction
ZP_01854760.1	NZ_ABCE01000016.1_73333_76125_+	G2L5	G2Lb	myRT prediction
ZP_01851752.1	ABCE01000001.1_117632_117919_-	G2L5	CRISPR-like	myRT prediction
EKP98429.1	CP006690.1_1914333_1915748_-	UG2	UG2	myRT prediction
CCC73043.1	HE576794.1_939599_941005_+	UG2	UG2	myRT prediction
AEG09910.1	CP002767.1_716105_717388_	UG2	UG2	myRT prediction
AEW72297.1	CP002886.1_903224_904396_+	UG3	UG3	myRT prediction
ACD38707.1	EU595736.1_18886_20160_+	UG3	UG3	myRT prediction
AHE72406.1	CP006580.1_34213_36348_-	UG4	UG4	myRT prediction
AGI74246.1	CP003742.1_4621630_4622688_+	UG4	UG4	myRT prediction
CDI94624.1	HG530068.1_6882792_6885812_+	UG5	UG5	myRT prediction
AHM47056.1	CP007393.1_928229_931438_-	UG5	UG5	myRT prediction
ACU61669.1	CP001699.1_5262939_5266187_+	UG6	UG6	myRT prediction
AHL77683.1	CP007441.1_3859031_3861010_-	UG8	UG8	myRT prediction
AEN62840.1	CP003026.1_82890_84845_-	UG8	UG8	myRT prediction
AFG37103.1	CP003282.1_1170484_1172610_-	UG8	UG8	myRT prediction
ADI30165.1	CP002056.1_2016134_2017783_-	UG9	UG9	myRT prediction
AGA65410.1	CP002873.1_44531_46162_+	UG14	UG14	myRT prediction

* We note that AFY43443.1 was labeled as a G2L1/G2L2 RT in [2], but the authors reported that the RTs classified as G2L1 and G2L2 are associated with *cas1* genes of CRISPR/Cas loci. G2L1 and G2L2 were proposed originally in [5], and in Toro et al [1], they were renamed as CRISPR-RT because of their association with the CRISPR-Cas systems.

Supplementary Table 4: Evaluation of myRT on the collection of retron RTs from [6], all of which were predicted as retron RT by myRT.

Known Retron	Genome	RT coordinates	Identity%[&]	myRT results
EC48[a]	*Escherichia Coli* DE147	LFQP01000005.1_154506_155696_-	50	myRT prediction
EC67[a]	*Escherichia coli* S10	CP010229.1_4712073_4713833_-	61	myRT prediction
EC73[a]	*Escherichia coli* M10	CP010200.1_2393178_2394128_+	36	myRT prediction
Ec78[a]	*Escherichia coli* 102598	JHRW01000018.1_27622_28557_+	49	myRT prediction
EC83[a]	*Escherichia coli* 05-2753	CXYK01000012.1_74586_75524_+	47	myRT prediction
Mx65	*Myxococcus xanthus* DSM 16526	FNOH01000027.1_37959_39242_+	53	myRT prediction
Eco8[a]	*Escherichia coli* 200499	CYGJ01000003.1_369367_370491_+	47	myRT prediction
Se72	*Salmonella enterica*[b]	AMMS01000284.1_2640_3671_-	49	myRT prediction
Vc137[a]	*Vibrio cholerae* 2012EL-1759	JNEW01000012.1_609188_610135_+	49	myRT prediction
Vp96	*Vibrio parahaemolyticus* S119	AWJG01000250.1_32_1054_+	49	myRT prediction
YF79	*Yersinia frederiksenii* ATCC 33641	KN150731.1_1692670_1693602_-	50	myRT prediction

[a] These retrons function as anti-phage defense systems. [b] Salmonella enterica enterica sv. Heidelberg 579083-10. [&] This column lists the highest sequence identity between the predicted RT and the RVT_1 domains used to build the HMMs for the different classes of RTs.

Supplementary Table 5: Distribution of different classes of RT in complete and draft genomes*

Index	RT Class	In Complete Genomes	In Draft Genomes	Sum	%
1	AbiA	38	262	300	0.1
2	AbiK	112	3027	3139	1.09
3	AbiP2	226	2826	3052	1.06
4	CRISPR (CRISPRs RT w/ Cas genes)	142	2021	2163	0.75
5	CRISPR-like (CRISPR RTs w/o Cas genes)	73	1961	2034	0.71
	CRISPR-other (Other RTs w/ Cas genes)	40	279	319	0.11
6	DGRs	459	16064	16523	5.76
7	G2L	167	23	190	0.07
8	G2Lb	15	9	24	0.01
9	G2Lc	4	19	23	0.01
10	G2L4	28	514	542	0.19
11	GII (Group II introns)	18848	162783	181631	63.31
12	Retrons	2973	30825	33798	11.78
13	UG1	71	682	753	0.26
14	UG2	399	3077	3476	1.21
15	UG3	199	1644	1843	0.64
16	UG4	228	2296	2524	0.88
17	UG5	213	2691	2904	1.01
18	UG6	101	71	172	0.06
19	UG7	151	1292	1443	0.5
20	UG8	215	2282	2497	0.87
21	UG9	31	365	396	0.14
22	UG10	28	207	235	0.08
23	UG11	119	3564	3683	1.28
24	UG12	94	728	822	0.29
25	UG13	20	271	291	0.1
26	UG14	38	208	246	0.09
27	UG15	82	1098	1180	0.41
28	UG16	20	243	263	0.09
29	UG17	177	2023	2200	0.77
30	UG18	58	196	254	0.09
31	UG19	15	242	257	0.09
32	UG20	9	73	82	0.03
33	UG21	2	27	29	0.01
34	UG22	16	66	82	0.03
35	UG23	21	70	91	0.03
36	UG24	13	144	157	0.05
37	UG25	10	102	112	0.04
38	UG26	5	823	828	0.29
39	UG27	12	424	436	0.15
40	UG28	47	352	399	0.14
41	UG28b	9	230	239	0.08
42	UNC	78	2329	2407	0.84
	Sum	25566	248154	273720	100

* As of 11/01/2021

Supplementary Table 6: myRT results for individual genomes

Accession	Genome/Plasmid	myRT results
CP005935.1	*Bacillus thuringiensis* YBT-1518	myRT prediction
BA000019.2	*Nostoc sp.* PCC 7120	myRT prediction
AP009552.1	*Microcystis aeruginosa* NIES-843	myRT prediction
U17233.3	*Lactococcus lactis* plasmid pTR2030	myRT prediction
U35629.2	*Lactococcus lactis* plasmid pSRQ800	myRT prediction
CP000247.1	*Escherichia coli* 536	myRT prediction
JGYE01000047.1	*Salmonella enterica* subsp. enterica serovar 9,12:l,v:- str. 94293	myRT prediction
CP004141.1	*Edwardsiella piscicida* C07-087	myRT prediction
CP002114.2	*Staphylococcus aureus* subsp. aureus JKD6159	myRT prediction
CP005935.1	*Bacillus thuringiensis* YBT-1518	myRT prediction
CP000703.1	*Staphylococcus aureus* subsp. aureus JH9	myRT prediction

Supplementary Table 7: Potential improvements of RT classification by using phylogenetic information

Accession number	Gene coordinates	hmmscan	pplacer	genomic neighborhood	myRT results
-	KB901875.1_2019009_2019602_+	CRISPR/**GII**	GII	GIIM	myRT prediction
ACN15726.1	CP001087.1_3000453_3001124_-	DGRs/**GII**	GII	GII	myRT prediction
CAE80471.1	BX842601.2_2599160_2603878_-	UG19/**UG10**/GII	UG10	DNA_primase_S	myRT prediction
QHQ68693.1	CP047142.1_1856232_1858199_+	UG12/**AbiK**/AbiP2	AbiK	Abi_2, Glyco_hydro_68	myRT prediction
QHQ68693.1	CP047142.1_1856232_1858199_+	DGRs/CRISPR/**UG6**	UG6	group_II_RT_mat (fused)	myRT prediction
BAZ36932.1	AP018280.1_22270_22950_+	DGRs/**GII**	GII	McrA	myRT prediction
-	KB901877.1_221182_221652_-	**GII**/UG4	GII	GIIM	myRT prediction
ABW09889.1	CP000820.1_498987_499841_+	DGRs/**GII**	GII	INT_RitC_C_like	myRT prediction
AHX61471.1	CP007567.1_2408799_2408972_+	**Retrons**/UG24/DGRs	Retrons	zf-IS66	myRT prediction
AUJ27137.1	CP015444.1_135192_137216_+	**AbiK**/UG12/UG15	AbiK	KAP_NTPase	myRT prediction
AUI76834.1	CP015498.1_1856458_1858044_-	textbfAbiK/UG12/UG15	AbiK	Abi_2, GlpR	myRT prediction
AZA22328.1	CP031016.1_1912675_1914699_+	**AbiK**/UG12/UG15	AbiK	AbiH	myRT prediction
-	KB901876.1_1554_2123_+	**GII**/DGRs	GII	GIIM	myRT prediction
ANU66363.2	CP015403.2_1765974_1766285_-	DGRs/**GII**	GII	GIIM	myRT prediction

Supplementary Table 8: myRT results for different metagenomes

Metagenome	Source	myRT results	myDGR results
ERR248260	Chicken	myRT prediction	myDGR prediction
ERR248261	Cow	myRT prediction	myDGR prediction
ERR248262	Human	myRT prediction	myDGR prediction
ERR248263	Pig	myRT prediction	myDGR prediction
ERR1135178	Pig	myRT prediction	myDGR prediction
ERR1135179	Pig	myRT prediction	myDGR prediction
ERR1135180	Pig	myRT prediction	myDGR prediction
ERR1135181	Pig	myRT prediction	myDGR prediction

Supplementary Table 9: Frequent domains in the genomic neighborhood of previously labeled RTs

RT class	Frequent neighbors
CRISPR-Cas RTs	Cas1 and Cas2
DGRs RTs	Avd_like, YfmG, and DUF1566
GII RTs	GIMM and RVT_N
UG17 RTs	SLATT_5
UG3 RTs	UG8 RTs
UG8 RTs	UG3 RTs
UG9 RTs	PRK14975 *
UG10 RTs	AE_Prim_S_like (appended)

*PRK14975: bifunctional 3'-5' exonuclease/DNA polymerase

Supplementary Table 10: Plasmids harbouring IMP-4 and IMP-26* [7, 8]

Accession NO.	Plasmid	Carbapenemase	Source	Length(bp)	myRT results
KM977631.1	pIMP-1495	IMP-4	*Klebsiella Pneumoniae*	50742	myRT prediction
KT982615.1	pIMP-FS1505	IMP-4	*Escherichia coli*	54449	myRT prediction
KT989598.1	pIMP-SH1506	IMP-4	*Enterobacter cloacae*	54669	myRT prediction
KU051708.1	pIMP-SZ1501	IMP-4	*Klebsiella Pneumoniae*	51469	myRT prediction
KU051710.1	pIMP-FJ1503	IMP-4	*Citrobacter freundii*	50546	myRT prediction
CP028486.1	p3	IMP-4	*Escherichia coli*	52864	myRT prediction
KU862632.1	pIMP-KP1495	IMP-4	*Klebsiella Pneumoniae*	51591	myRT prediction
KX711879.1	P378-IMP	IMP-4	*Pseudomonas aeruginosa*	51207	myRT prediction
KY913900.1	p4-IPM	IMP-4	*Klebsiella oxytoca*	61680	myRT prediction
MF344559.1	p128379-IMP	IMP-4	*Enterobacter hormaechei*	42279	myRT prediction
CP033103.1	pEHZJ1	IMP-26	*Enterobacter hormaechei*	343918	myRT prediction
MH399264.1	pIMP26	IMP-26	*Enterobacter cloacae*	329420	myRT prediction

More examples are available at myRT-blaIMP

complete genomes			
**Domains**	**Number of occurrences (%)**	**Specificity score**	**Co-occurring RTs**
RVT-UG28b	9 (100%)	1 (9/9)	link | piechart
VirE_N (pfam08800)	7 (78%)	0.636 (7/11)	link | piechart
trehalose_TreZ (TIGR02402)	1 (11%)	1 (1/1)	link | piechart
Tra8 (COG2826)	1 (11%)	0.004 (1/274)	link | piechart
TPR (smart00028)*2	1 (11%)	0.023 (1/44)	link | piechart
TPR (smart00028)	1 (11%)	0.008 (1/126)	link | piechart
RelE (pfam06296)	1 (11%)	0.04 (1/25)	link | piechart
PRK09358 (adenosine deaminase; Provisional)*2	1 (11%)	0.5 (1/2)	link | piechart
PRK09358 (adenosine deaminase; Provisional)	1 (11%)	0.5 (1/2)	link | piechart
PriCT_2 (pfam08707)	1 (11%)	0.2 (1/5)	link | piechart
polC (TIGR00354)	1 (11%)	0.045 (1/22)	link | piechart
Phage_int_SAM_5 (pfam13102)	1 (11%)	0.016 (1/64)	link | piechart
Peptidase_M50B (pfam13398)	1 (11%)	1 (1/1)	link | piechart
PemK_toxin (pfam02452)	1 (11%)	0.053 (1/19)	link | piechart
pcm (PRK00312)	1 (11%)	0.25 (1/4)	link | piechart

draft genomes			
**Domains**	**Number of occurrences (%)**	**Specificity score**	**Co-occurring RTs**
RVT-UG28b	230 (100%)	1 (230/230)	link | piechart
VirE_N (pfam08800)	102 (44%)	0.935 (101/108)	link | piechart
PriCT_2 (pfam08707)	46 (20%)	0.807 (46/57)	link | piechart
DUF932 (pfam06067)	14 (6%)	0.028 (14/509)	link | piechart
SMC_N (pfam02463)	9 (4%)	0.184 (9/49)	link | piechart
RVT-UG28b*2	9 (4%)	1 (9/9)	link | piechart
AE_Prim_S_like (cl01287)	8 (3%)	0.047 (8/170)	link | piechart
DUF262 (pfam03235)	7 (3%)	0.016 (7/427)	link | piechart
SMC_N (pfam02463)*2	6 (3%)	0.667 (6/9)	link | piechart
Por_Secre_tail (TIGR04183)	6 (3%)	0.029 (6/206)	link | piechart
HTH_XRE (smart00530)	6 (3%)	0.001 (6/4863)	link | piechart
COG4849 (Predicted nucleotidyltransferase)	6 (3%)	0.6 (6/10)	link | piechart
COG4551 (Predicted protein tyrosine phosphatase)	6 (3%)	0.857 (6/7)	link | piechart
CBM6_cellulase-like (cd04080)	6 (3%)	0.462 (6/13)	link | piechart
Big_7 (pfam17957)	6 (3%)	0.25 (6/24)	link | piechart

Supplementary Figure 1: Number of occurrences of domains in UG28b RTs* and their genomic neighborhood in complete and draft bacterial genomes (see the table at the myRT website).

Distribution of RTs co-occuring with PRK10473 in complete genomes:

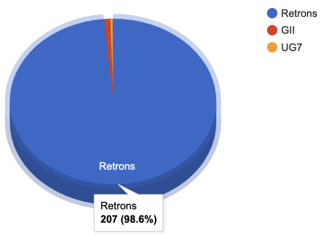

Found **PRK10473** domain in the genomic neighborhood of RTs in complete genome(s) shown below (complete/draft | GCA-id | Species name (70 characters max)| RT | RT class | myRT prediction):

- complete |GCA_013811445.1_ASM1381144v1 | Klebsiella pneumoniae strain RHB39-C10 chromosome | CP057066.1_5133549_5135087_+ | **RVT-UG7** | (View)
- complete |GCA_000006945.2_ASM694v2 | Salmonella enterica subsp. enterica serovar Typhimurium str. LT2 | AE006468.2_4052419_4053288_+ | **RVT-Retrons** | (View)
- complete |GCA_000022165.1_ASM2216v1 | Salmonella enterica subsp. enterica serovar Typhimurium str. 14028S | CP001363.1_4066033_4066968_+ | **RVT-Retrons** | (View)
- complete |GCA_000027025.1_ASM2702v1 | Salmonella enterica subsp. enterica serovar Typhimurium str. D23580 c | FN424405.1_4075282_4076151_+ | **RVT-Retrons** | (View)
- complete |GCA_000188735.1_ASM18873v1 | Salmonella enterica subsp. enterica serovar Typhimurium str. ST4/74 | CP002487.1_4073652_4074587_+ | **RVT-Retrons** | (View)
- complete |GCA_000188955.5_ASM18895v5 | Salmonella enterica subsp. enterica serovar Montevideo str. 507440-20 | CP007530.1_3626350_3627285_+ | **RVT-Retrons** | (View)
- complete |GCA_000210855.2_ASM21085v2 | Salmonella enterica subsp. enterica serovar Typhimurium SL1344 comple | FQ312003.1_4073651_4074586_+ | **RVT-Retrons** | (View)
- complete |GCA_000213635.1_ASM21363v1 | Salmonella enterica subsp. enterica serovar Typhimurium str. UK-1 | CP002614.1_4013706_4014575_+ | **RVT-Retrons** | (View)
- complete |GCA_000238455.3_ASM23845v3 | Salmonella enterica subsp. enterica serovar Montevideo str. 42N chrom | CP037893.1_31172_32041_- | **RVT-Retrons** | (View)
- complete |GCA_000252875.1_ASM25287v1 | Salmonella enterica subsp. enterica serovar Typhimurium str. 798 | CP003386.1_4072487_4073422_+ | **RVT-Retrons** | (View)
- complete |GCA_000271905.2_ASM27190v2 | Salmonella enterica subsp. enterica serovar Newport str. Levine 15 | CP015924.1_4877049_4877981_+ | **RVT-Retrons** | (View)
- complete |GCA_000283735.1_ASM28373v1 | Salmonella enterica subsp. enterica serovar Typhimurium str. T000240 | AP011957.1_4157452_4158387_+ | **RVT-Retrons** | (View)
- complete |GCA_000380325.1_ASM38032v1 | Salmonella enterica subsp. enterica serovar Typhimurium str. U288 | CP003836.1_4047237_4048172_+ | **RVT-Retrons** | (View)
- complete |GCA_000430145.3_ASM43014v3 | Salmonella enterica subsp. enterica serovar Typhimurium var. 5- str. | CP006048.1_4374152_4375087_- | **RVT-Retrons** | (View)
- complete |GCA_000486345.2_ASM48634v2 | Salmonella enterica subsp. enterica serovar Typhimurium str. SARA13 c | CP017728.1_4811560_4812495_- | **RVT-Retrons** | (View)
- complete |GCA_000486815.2_ASM48681v2 | Salmonella enterica subsp. enterica serovar Oranienburg str. 0250 str | CP019197.1_4640564_4641499_- | **RVT-Retrons** | (View)

Supplementary Figure 2: PRK10473 domains found in the genomic neighborhood of RTs in complete genomes (see details at the myRT website).

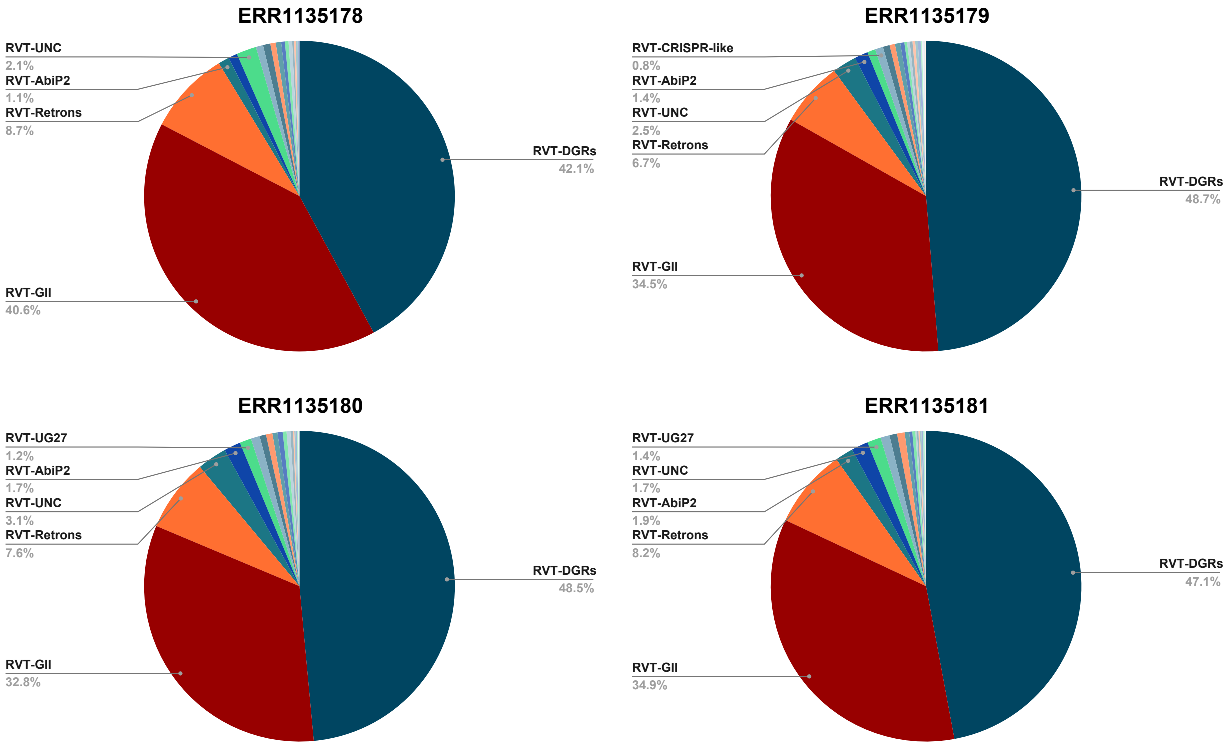

Supplementary Figure 3: Distribution of different RT classes in pig gut metagenomes from [9].

References

[1] Nicolás Toro and Rafael Nisa-Martínez. Comprehensive phylogenetic analysis of bacterial reverse transcriptases. *PLoS One*, 9(11):e114083, 2014. [PubMed:25423096] [PubMed Central:PMC4244168] [doi:10.1371/journal.pone.0114083].

[2] Steven Zimmerly and Li Wu. An unexplored diversity of reverse transcriptases in bacteria. *Mobile DNA III*, pages 1253–1269, 2015. [PubMed:26104699] [doi:10.1128/microbiolspec.MDNA3-0058-2014].

[3] Thomas Schillinger, Mohamed Lisfi, Jingyun Chi, John Cullum, and Nora Zingler. Analysis of a comprehensive dataset of diversity generating retroelements generated by the program digref. *BMC genomics*, 13(1):430, 2012. [PubMed:22928525] [PubMed Central:PMC3521204] [doi:10.1186/1471-2164-13-430].

[4] Sukrit Silas, Kira S Makarova, Sergey Shmakov, David Páez-Espino, Georg Mohr, Yi Liu, Michelle Davison, Simon Roux, Siddharth R Krishnamurthy, Becky Xu Hua Fu, et al. On the origin of reverse transcriptase-using crispr-cas systems and their hyperdiverse, enigmatic spacer repertoires. *MBio*, 8(4), 2017. [PubMed:28698278] [PubMed Central:PMC5513706] [doi:10.1128/mBio.00897-17].

[5] Dawn M Simon and Steven Zimmerly. A diversity of uncharacterized reverse transcriptases in bacteria. *Nucleic acids research*, 36(22):7219–7229, 2008. [PubMed:19004871] [PubMed Central:PMC2602772] [doi:10.1093/nar/gkn867].

[6] Anna J Simon, Andrew D Ellington, and Ilya J Finkelstein. Retrons and their applications in genome engineering. *Nucleic acids research*, 47(21):11007–11019, 2019. [PubMed:31598685] [PubMed Central:PMC6868368] [doi:10.1093/nar/gkz865].

[7] Yingying Hao, Chunhong Shao, Xu Geng, Yuanyuan Bai, Yan Jin, and Zhiming Lu. Genotypic and phenotypic characterization of clinical escherichia coli sequence type 405 carrying incn2 plasmid harboring blandm-1. *Frontiers in microbiology*, 10:788, 2019. [PubMed:31105653] [PubMed Central:PMC6499153] [doi:10.3389/fmicb.2019.00788].

[8] Jian-Jun Gou, Na Liu, Li-Hua Guo, Hao Xu, Tao Lv, Xiao Yu, Yun-Bo Chen, Xiao-Bing Guo, Yu-Ting Rao, and Bei-Wen Zheng. Carbapenem-resistant enterobacter hormaechei st1103 with imp-26 carbapenemase and esbl gene blashv-178. *Infection and Drug Resistance*, 13:597, 2020. [PubMed:32110070] [PubMed Central:PMC7039083] [doi:10.2147/IDR.S232514].

[9] Liang Xiao, Jordi Estellé, Pia Kiilerich, Yuliaxis Ramayo-Caldas, Zhongkui Xia, Qiang Feng, Suisha Liang, Anni Øyan Pedersen, Niels Jørgen Kjeldsen, Chuan Liu, et al. A reference gene catalogue of the pig gut microbiome. *Nature microbiology*, 1(12):1–6, 2016. [PubMed:27643971] [doi:10.1038/nmicrobiol.2016.161].
